# Low dose aspirin and low-molecular-weight heparin in the treatment of pregnant Libyan women with recurrent miscarriage

**DOI:** 10.1186/1756-0500-7-23

**Published:** 2014-01-09

**Authors:** Mohamed O Elmahashi, Aisha M Elbareg, Fathi M Essadi, Bashur M Ashur, Ishag Adam

**Affiliations:** 1 Department of Obstetrics and Gynaecology, Misurata Central Hospital, P.O. Box: 2472, Misurata, Libya; 2 Department of Paediatrics, Misurata Central Hospital, P.O. Box: 2472, Misurata, Libya; 3 Department of Obstetrics and Gynecology, University of Khartoum, Khartoum, Sudan

**Keywords:** Low-molecular-weight heparin, Enoxaparin, Low dose aspirin, Recurrent miscarriage

## Abstract

**Background:**

Recurrent miscarriage is a major women’s health problem. Aspirin and heparin have been shown to have potentially beneficial effects on trophoblast implantation. However, few published data on this issue are available from developing countries.

**Methods:**

An open clinical trial was conducted at the Department of Obstetrics and Gynecology at Misurata Teaching Hospital in Libya from January 2009 to December 2010 to investigate the effects of treatment with low dose aspirin (LDA) versus treatment with low-molecular-weight-heparin (LMWH) in combination with LDA on patients with a history of recurrent miscarriages. A total of 150 women were enrolled in the study. Women were eligible for the study if they had a history of three or more consecutive miscarriages. Participants were randomly assigned to receive either LDA (75 mg daily) alone or a combination of LDA and LMWH (75 women per treatment group). The primary outcomes were the rate of miscarriages and live births for each group.

**Results:**

Compared with the group who received LDA alone, the combination group had a significantly lower number of miscarriages (22/75 [29%] vs. 43/75 [47%], P < 0.001) and had a significantly higher number of live births (53/75 [71%] vs. 32/75 [42%], P < 0.001). Two preterm infants in the LDA group and three in the combination group were admitted to the neonatal intensive care unit. There were no significant differences in the mean (SD) birth weights of neonates born in either group (2955.4 ± 560 vs. 3050 ± 540 g for the LDA and combination groups, respectively, P = 0.444). There were no congenital abnormalities detected in either group.

**Conclusion:**

The combination of LDA and LMWH is better than LDA alone for the maintenance of pregnancy in patients with recurrent first trimester miscarriage.

**Trial registration:**

NCT01917799

## Background

Recurrent pregnancy loss is a major women's health issue; 1% to 2% of women of reproductive age have experienced three or more successive pregnancy losses, and approximately 5% have lost at least two successive pregnancies [1]. A small proportion of these losses are associated with identifiable causes in the mother or fetus, but the underlying cause in most cases of recurrent pregnancy loss remains unknown. There is evidence to suggest that successful pregnancy outcome depends on the development and maintenance of adequate utero-placental circulation, and that the hypercoagulability associated with thrombophilia might result in recurrent miscarriages [2-4]. However, there is no clear consensus on what constitutes appropriate thrombophilia testing or, indeed, a standardized laboratory method for thrombophilia assessment.

Because of the potential involvement of thrombophilia in recurrent miscarriage, the use of antithrombotic agents has been suggested as a potential means of increasing the live birth rates in subsequent pregnancies in women with either inherited thrombophilia or unexplained recurrent miscarriages [5-7]. Preliminary data examining pregnancy success with the use of antithrombotic therapy in women with heritable thrombophilia are inconclusive [8,9]. However, because two successive pregnancy losses are relatively common and distressing, and there are no other effective treatments, antithrombotic therapy is often prescribed for these women. Although low-molecular weight heparin (LMWH) and low dose aspirin (LDA) are generally considered safe [10,11], there is no direct evidence of their efficacy for miscarriage prevention. As a result, there have been repeated calls for randomized trials in this area, particularly a comparison of anticoagulant treatment with no pharmacological intervention [9,12].

Heparin has been shown to have potentially beneficial effects on trophoblast implantation [13,14] and influence trophoblast apoptosis. To be beneficial, heparin may need to be given at the time of implantation. LMWHs are administered subcutaneously once a day. They have considerable theoretical benefit over unfractionated heparin (UFH) including better bioavailability, a longer plasma half-life [15], more predictable pharmacokinetics and pharmacodynamics [16], and less potential to cause osteoporosis [17]. LMWH is also less likely to induce thrombocytopenia [18]. LMWH inhibits factor Xa more effectively than factor IIa to produce its antithrombotic effect [19]. LMWH does not cross the placenta and is safe for the fetus [20,21].

Aspirin is increasingly used to reduce the risk of miscarriage and improve pregnancy outcome in women who have suffered recurrent miscarriage. An important factor controlling tissue perfusion is the equilibrium between thromboxane A2 (in addition to its platelet aggregating properties, it also has a vasoconstrictor effect) and prostacyclin (has vasodilatory properties) [22]. The daily administration of LDA induces a shift in the balance away from thromboxane A2 and towards prostacyclin, leading to vasodilatation and enhanced blood flow [23]. The objective of this study was to compare the effects of LDA alone with the combination of LMWH and LDA in women with recurrent miscarriage.

## Methods

Women were eligible for this study if they had a history of three or more consecutive miscarriages. Women with a history of thromboembolism, systemic lupus erythematosis, uterine abnormalities, and multiple successful pregnancies were excluded. After obtaining informed consent, socio-demographic, obstetric, and medical data were gathered from the study participants using pre-tested questionnaires. The participants were randomly assigned to receive either LDA alone (LDA group) or a combination of LDA and LMWH (combination group) using computer generated numbers drawn from an envelope. Participants started taking 75 mg of LDA once daily as soon as their pregnancy was confirmed and fetal heart activity was detected by ultrasound. Women in the combination group also self-administered 0.4 mL/day of the LMWH Enoxaparin. Follow-up examinations were conducted in the antenatal care clinic and all participants received routine iron and folic acid supplements. Participants were closely monitored until delivery, which was planned at 37 weeks of gestational age, unless otherwise indicated. Treatment was stopped at the time of miscarriage or when the pregnancy reached 34 weeks gestation. The outcome of all pregnancies was analyzed and all neonates were examined by a pediatrician shortly after delivery.

### Statistics

Data analyses were performed using SPSS for Windows. Means (SD) and proportions were compared between the two groups using Student’s t-tests and chi square tests, respectively. Between-group differences were regarded as significant when P < 0.05.

### Ethics

This study was approved by the Ethical Committee of the Faculty of Medicine at Misurata University, Libya. Written informed consent was obtained from all study participants.

## Results

A total of 150 women were enrolled in the study (75 per treatment group). There were no significant differences between the two groups in their age, parity, number of previous miscarriages, gestational age, and body mass index at the time of enrollment (Table 1).

**Table 1:** Sociodemographic characteristics of the women who received low dose aspirin and low dose aspirin and low molecular weight heparin combination therapy

**Variable**	**LDA (n = 75)**	**LDA and LMWH (n = 75)**	**P**
Age, years	26.5 (2.7)	27.3 (4.8)	0.210
Parity	0.8 (1.3)	1.1 (1.3)	0.152
Body mass index	26.2 (5.1)	25.6 (4.7)	0.545
Previous miscarriage	4.1 (2.1)	3.9 (3.2)	0.651
Gestational age at enrollment, week	8.1 (1.6)	8.3 (1.4)	0.416

The combination group had a significantly lower number of miscarriages (22 [29%] vs. 43 [47%], P < 0.001) and a significantly higher number of live births compared with the group that received LDA alone (53 [71%] vs. 32 [42%], P < 0.001). There were no significant differences in the number of preterm deliveries (< 37 weeks) between the two groups (13/53 [24.5%] vs. 7/32 [22.0%] for the LDA and combination groups, respectively, P = 0.789). Two women in the LDA group had pre-eclampsia and delivered at 36 weeks gestation. None of the women in either group developed a thromboembolic complication during pregnancy or puerperium.

Two neonates in the LDA and three neonates in the combination group were admitted to the neonatal unit because they were delivered preterm and needed observation and feeding. Birth weight did not significantly differ between the two groups, with a mean birth weight of 2955.4 ± 560 g in the LDA group and 3050 ± 540 g in the combination group (P =0.444). There were no congenital abnormalities detected in either group.

## Discussion

Our results indicate that treatment with a combination of LDA and LMWH leads to a significantly lower rate of miscarriages and higher rate of live births in pregnant women with a history of recurrent miscarriages than does LDA treatment alone. These findings are comparable with those of previous studies, which also concluded that the combination of heparin and aspirin is superior to aspirin alone in achieving higher rates of live births [24,25]. Another recent study also concluded that the use of LMWH is a safe and reliable treatment resulting in a high live birth rate and no maternal or fetal complications [26]. However, other measures that these women received, such as strict follow-up of all women, might have also affected the results because close follow-up has been reported to have a significant beneficial effect on pregnancy outcome in women with a history of recurrent miscarriages [27].

LDA may improve pregnancy outcome by irreversibly blocking the action of cyclo-oxygenase in platelets, thereby inhibiting platelet thromboxane synthesis and preventing thrombosis of the placental vasculature [28]. In addition to its anticoagulant action, heparin may act to reduce fetal loss by binding to phospholipids, thereby protecting trophoblast phospholipids from attack and promoting successful implantation in early pregnancy [29]. In our study, there was no difference in pregnancy outcome between the two treatment groups in the pregnancies that survived beyond 13 weeks gestation. By this time, the first wave of trophoblast invasion is complete and placentation is established. Therefore, our result suggests that heparin may indeed act to protect the developing trophoblast. However, a previous study showed that combination therapy with LMWH and aspirin failed to prevent late-pregnancy losses [24].

Despite the treatment, one quarter of the fetuses from the successful pregnancies were delivered prematurely. This confirms previous reports of a high incidence of pregnancy complications in patients with recurrent miscarriages, especially if phospholipid antibodies are detected [30-32], and emphasizes the need for close antenatal surveillance. Of the total deliveries, 24% were preterm. Most of these occurred spontaneously, but in some cases, labor was induced prematurely because of intrauterine growth retardation and preeclampsia. Five of these premature infants were admitted to the neonatal unit, but all of them were later discharged in good condition. The finding that most miscarriages occurred before 14 weeks of gestation confirms previous prospective observations in women with phospholipid antibodies who received no pharmacological treatment during pregnancy, and who were followed up from the time that they had a positive pregnancy test. Interestingly, Kaandorp *et al.* concluded in their recent review that there was no benefit of LDA over heparin treatment on the live-birth rate in women with a history of at least two miscarriages without apparent causes other than inherited thrombophilia [33].

In the current study, two women in the LDA group had pre-eclampsia. A link (perhaps through endothelial dysfunction and heptahelical G-protein-coupled receptors (GPCRs) has been postulated between pre-eclampsia and increased cardiovascular disease later in life and women with unexplained recurrent miscarriages who might be at increased cardiovascular risk [34,35]. The role of calcium/calmodulin-dependent kinase IV (CaMKIV) in blood pressure regulation (through the control of endothelial nitric oxide synthase activity), increased levels of G protein coupled receptor kinase and action of heparin by acting as a GRK inhibitor was observed [36,37].

Regarding maternal outcomes, LDA and LMWH are safe drugs, and were well tolerated in this study. Of those taking heparin, none developed thrombocytopenia or had symptomatic complications, apart from mild localized bruising at the injection site.

## Conclusion

The combination of LDA and LMWH is better than LDA alone for the maintenance of pregnancy in patients with recurrent first trimester miscarriages.

## Competing interests

The authors declare that they have no competing interests.

## Authors’ contributions

MOE and IA designed the study. AME, BMA, and FME conducted the clinical work. AME and IA conducted the statistical analyses. All authors approved the final manuscript.
